# Midlife aging and performance study (MAPS): evaluating biological aging through a physical capacity battery

**DOI:** 10.1007/s11357-025-01803-6

**Published:** 2025-08-05

**Authors:** Roy Tzemah-Shahar, Ilona Shapiro, Einat Kodesh, Merav Asher, Yechiel Friedlander, Hagit Hochner, Maayan Agmon

**Affiliations:** 1 School of Nursing, Faculty of Health and Social Welfare, University of Haifa, Haifa, Israel; 2 Epidemiology Unit, Hebrew University School of Public Health, Jerusalem, Israel; 3 Physical Therapy Department, Faculty of Health and Social Welfare, University of Haifa, Haifa, Israel

**Keywords:** Physical fitness, Exercise, Biological age, Accelerated aging

## Abstract

Aging is a risk factor for the development of chronic diseases; however, it is heterogeneous. Measuring aging in midlife, commonly done using laboratory markers and statistical methods for estimation of biological age (BA), cannot directly inform behavioral intervention goals aiming to promote healthier aging. The Midlife Aging and Performance Study (MAPS) examined the association between an extended, inclusive assessment of physical capacity (PC), as a behavioral marker of aging, and BA, estimated using the Klemera-Doubal method from 11 laboratory and physiological biomarkers, in 112 individuals aged 42–46 (47% women). PC was comprehensively measured by a battery covering five domains: muscle strength, endurance, balance, agility and flexibility. Better performance in strength, endurance, balance, and flexibility domains was correlated with younger BA (Pearson’s *r* 0.33–0.49, *p* < 0.001). A lower composite PC score based on all five domains was significantly associated with an accelerated aging state in which BA was greater than chronological age. In a logistic regression, a composite PC score had an odds ratio of 0.40 (95% CI 0.25–0.64), demonstrating each incremental rise in PC corresponds with a 60% odds reduction of being in an accelerated aging state. The proposed PC battery could be used as a functional behavioral assessment for aging state, relevant for population wide risk-screening assessments, communicating intervention goals, and as a means to evaluate temporal changes in health, independent of laboratory tests.

**Supplementary Information:**

The online version contains supplementary material available at 10.1007/s11357-025-01803-6.

## Introduction

Aging is a risk factor for the development of chronic diseases and functional decline, yet the aging process is highly diverse [1]. Biological age (BA) captures both between and within individual heterogeneity of aging by representing the overall body state [2], thus better estimating health-related status than chronological age (CA) alone [3]. Given the rising prevalence of age-related diseases [4], quantifying aging has become a frequent focus in research [5], and may assist in identifying individuals at risk for accelerated aging (where BA > CA), promoting personalized preventive interventions [6, 7]. Although there is no single gold standard for estimating BA, many studies use laboratory-based methodologies, such as molecular ageing clocks [8] that offer an acceptable quantification for different aspect of aging [9–11], while other studies quantify BA using a composition of laboratory and physiological markers [12–14]. Methods based on laboratory markers are costly, making them primarily suitable for research [15]. Furthermore, laboratory methods are less practical as a means to informing behavioral changes [16], such as promoting a more active lifestyle, leading to a better physical state and healthier aging process [17, 18].

Physical capacity (PC) [19], defined as a collection of functional markers associated with health and aging [20, 21], can serve as an alternative to the laboratory-based BA measurement, capturing individual’s health status by using behavioral markers of function [22, 23]. Although PC has been previously proposed as a means to quantify aging [24, 25], majority of available studies focus on muscular strength, or use a narrow testing battery [23], thus limiting our ability to effectively use PC markers in aging research. Published tests tend to ignore personal ability and sex-specific differences in performance [26, 27]. Moreover, the majority of available literature on function focuses on older adults, overshadowing the midlife period, a critical window of opportunity to facilitate a positive lifestyle change to improve the aging process [28]. Lastly, limitations of previous studies arise from the methods used in estimating BA, such as telomere length or specific laboratory markers that do not examine aging status comprehensively [23].

Filling these gaps is important for several reasons. First, PC is a broad concept, covering many distinct domains—muscular strength, endurance, flexibility, agility, and balance—corresponding with different ability requirements. Using a composite and inclusive measure of PC can provide an extensive representation of the body’s functional state [23] and available physical intrinsic capacity [29]. Second, to accommodate varying performance levels or possible sex-related differences [30], multiple tests with graded difficulty should be used when assessing PC. Moreover, midlife adults typically demonstrate relatively high physical ability yet remain understudied in aging research; using a graded approach in this age group can support enhanced detection of subtle inter-individual differences in PC that may relate to accelerated aging processes [2]. Lastly, by estimating BA using a composite measure based on laboratory and physiological markers [31] and comparing it with PC, the link between PC and aging state can be better understood. Developing PC measures for BA estimation that address these limitations can offer a practical and feasible alternative to the laboratory-based methodologies that is both community-accessible and informative for promoting positive health behavioral changes [32].

The Midlife Aging and Performance Study (MAPS) aims to examine the association between an extended, inclusive measure of PC—covering five functional domains—and BA in midlife, considering sex differences and leveraging graded levels of performance.

## Methods

This study comprises a subset of the Jerusalem Perinatal Study [33]; participants from the most recent follow-up point, at ages 42–45 (conducted between 2016 and 2021) [34], that were willing to take part in an extension study were included in MAPS. The inclusion criterion was a subjective ability to engage in leisure time exercise. Exclusion criteria were (a) acute illness (in the week prior to the testing day); (b) acute musculoskeletal complaint (e.g., acute low back pain) or known chronic condition (e.g., cardiovascular disease) subjectively limiting participation in physical exercise; and (c) use of medications that might influence physical performance or pose a risk when performing exercises (e.g., non-steroid anti-inflammatory drugs) on testing day. Each participant received general information on the study aims and provided signed informed consent prior to testing. The Ethics Committee of the Faculty of Social Welfare and Health Sciences at the University of Haifa (#407/20) and the Hadassah Hospital Institutional Review Board (#10–01.04.05) provided ethical approval for this study.

### BA estimation

Eleven biomarkers were used to estimate BA: six fasting blood measures (glucose, insulin, high-density lipoproteins, low-density lipoproteins, triglycerides, total cholesterol) and five physiological markers (body mass index, diastolic resting blood pressure, systolic resting blood pressure, resting heart rate, and waist circumference) were added to the Klemera-Doubal method (KDM) [12] algorithm using the BioAge R package [31] to calculate BA. KDM estimates BA by weighing each marker according to its correlation with chronological age in training cohort data [12]. BioAge uses the third United States Health and Nutrition Examination Surveys (NHANES) [35] dataset as the training cohort. Thus, participants’ BA was estimated by determining the corresponding CA at which an individual’s biomarkers would be considered normal in the training dataset. See Supplementary Table S1 for additional information.

### Physical capacity measurements

**Fig. 1:**
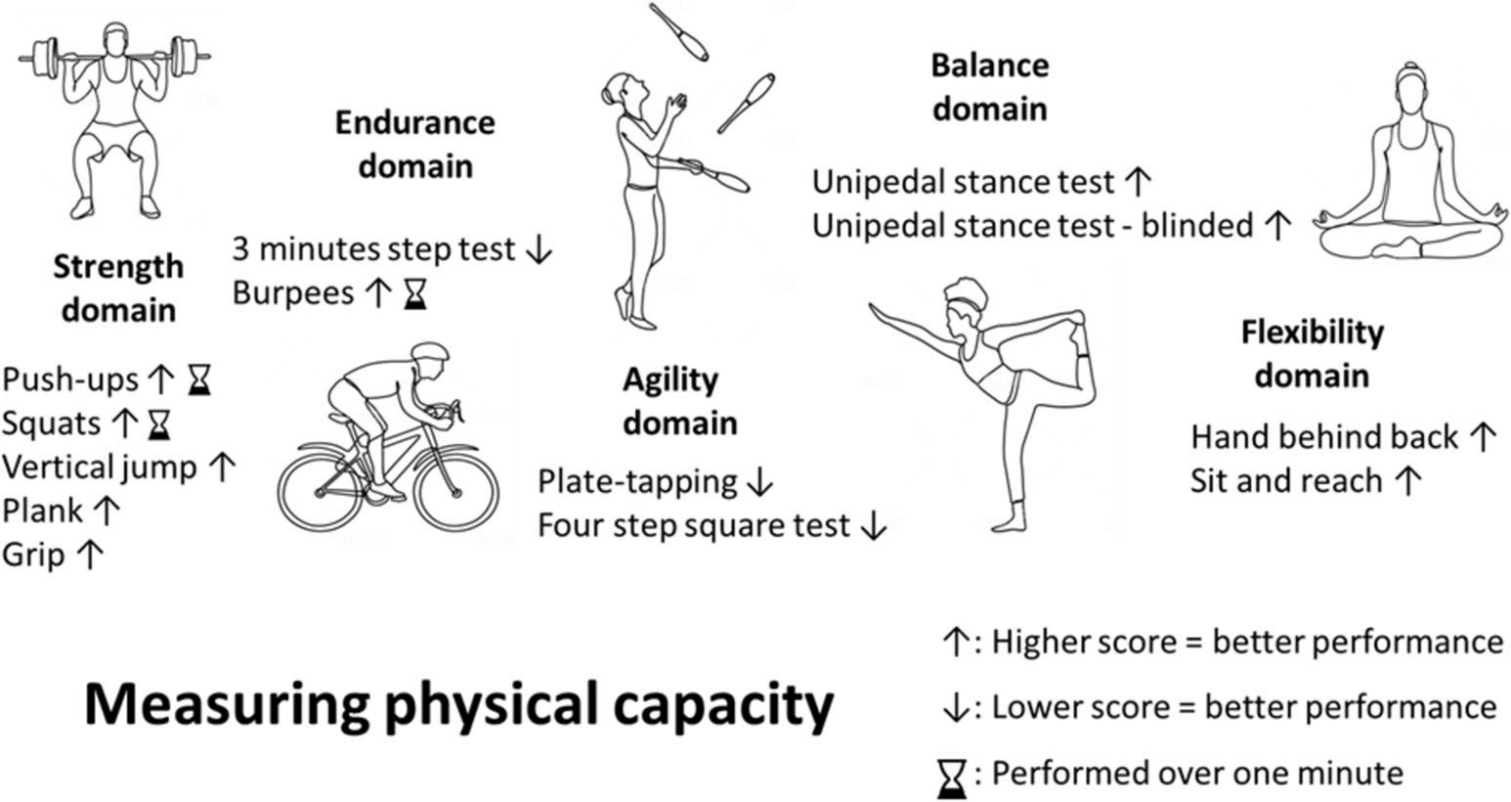
Physical capacity tests by domain

Measuring PC requires testing individuals’ abilities to perform tasks that capture different aspects of physical function; there are a range of fitness tests suitable for measuring PC, accounting for age and sex differences and PC domains of interest [36–38]. Five main PC domains were identified and previously studied in the context of BA (strength, endurance, agility, balance, flexibility); here, we measured these domains using 13 different tests (Fig. 1). Specific test selection was based on suitability to a home environment and, where possible, previous validation in a similar population. A detailed list of performed tests and testing procedure is provided in Supplementary Table S1. Calculations of domain specific and composite PC (ΣPC) scores were based on standardized ranking and percentile calculation, performed separately for each sex, like a previously used method for calculating ΣPC [39]. First, each test’s results were categorized based on percentiles on a scale of 0 to 1 (1 corresponding with best performance); domain scores were derived by averaging the standardized results of included tests (Fig. 1). We calculated ΣPC scores by summing the five domains, for a total score ranging from 0 to 5. See Supplementary Table S2 for additional information on standardization processes. Subjective perceived exertion in the entire testing session was also recorded, by asking each participant to rate his or her exertion once the session was over, on a visual analogue scale (VAS) ranging from 0 to 10 (10 corresponding with maximal exertion) [40].

### Data analysis

Analysis of results was conducted using IBM SPSS v.27.0.0.0. We compared MAPS cohort and Jerusalem Perinatal Study sub-cohort to identify significant differences. The associations between the different standardized tests or PC domain scores and BA were examined using Pearson’s partial correlation, controlling for chronological age. Significantly correlated domains were then included in a linear regression model to identify the independent contribution of each domain to BA, while controlling for chronological age. Lastly, we estimated the contribution of ΣPC to being in an accelerate aging state: The differences between BA and CA were dichotomized (∆Age > 0 representing accelerated aging state, ∆Age ≤ 0, normal to slower aging), and a logistic regression was used to assess the effect (odds ratio) of ΣPC on accelerated aging state, controlling for CA. Investigation of sex-specific differences in PC performance was done by comparing tests results scores prior to the ranking procedure, tests completion rates, and reported exertion levels of the entire testing procedure (examined using *t*-tests or Mann–Whitney tests, as appropriate).

## Results

### Participant characteristics

**Fig. 2:**
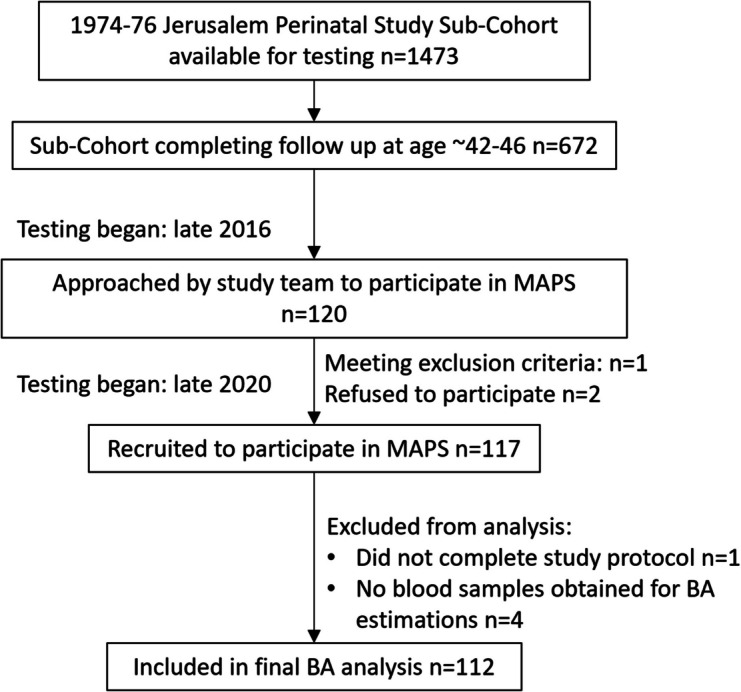
Study procedures flow chart

The latest Jerusalem Perinatal Study follow-up at age ~ 42–46 years included a total of *n* = 672 individuals from the complete original cohort. Data collection began in late 2016, while collection for MAPS began in November 2020 and was completed by November 2021, when the pool of volunteers was exhausted. A total of 120 individuals were approached, and 117 consented to participate in MAPS (see Fig. 2). One man was excluded due to subjective complaints of acute lower back pain limiting his ability to participate in physical exercise, and two women refused participation. Of the remaining 117 participants, five were omitted from the final analysis: one man was unable to complete the testing battery due to an adverse response to the physical effort, resulting in termination of testing (the participant had complaints of nausea and chest pain and was referred to an emergency room, resulting in coronary artery catheterizations for previously undiagnosed coronary arteries disease), and four participants did not provide the blood samples needed for BA estimation. Demographics, anthropometric data, and BA estimation of the 112 participants (53 women, 47%) included in the analysis, and comparisons with the remaining Jerusalem Perinatal Study sub-cohort tested are presented in Table 1. This comparison revealed that individuals consenting to participate in MAPS were slightly older (the age differences were associated with majority of Jerusalem Perinatal Study participants being examined before COVID-19 pandemic, while MAPS participants were examined later, following the first pandemic waves), with participating men having a lower BMI and smoking less than the men in the overall sub-cohort.

**Table 1:** MAPS cohort characteristics and selection bias analysis

	MAPS (mean ± SD) *N* = 112		Jerusalem Perinatal Study sub-cohort (mean ± SD)^1^, *N* = 560	
	Women (*n* = 53)	Men (*n* = 59)	Women (*n* = 285)	Men (*n* = 275)
CA (years)^2^	44.5 ± 1.4	44.5 ± 1.4	42.9 ± 1.1	43.0 ± 1.2
BMI	26.8 ± 4.6	27.0 ± 3.9	27.6 ± 6.0	28.5 ± 4.6
Waist circumference (cm)^3^	85.3 ± 11.8	96.5 ± 11.1	85.9 ± 12.6	98.3 ± 12.0
Education (years)	15.9 ± 1.9	16.6 ± 3.4	15.3 ± 2.6	15.9 ± 4.2
Smoking (pack years)	1.3 ± 3.3	1.9 ± 3.1	1.2 ± 3.5	4.5 ± 6.6

*CA* chronological age, *MAPS* Midlife Aging and Performance Study, *BMI* body mass index, *SD* standard deviation

^1^Comparisons between male and female participants included in MAPS and the remaining sub-cohort revealed significant differences in age for both sexes, and differences in BMI and smoking for men only (included men had lower BMI and lower smoking pack years). The observed age difference was associated with the time difference between Jerusalem Perinatal Study follow-up and MAPS initiation.

^2^Age at time of blood sample collection.

^3^Women/men statistically different in MAPS and the complete sub-cohort, T-test, *p* < 0.05.

### BA estimation and PC testing battery

In the MAPS cohort, BA ranged from 31.3 to 51.3 years with accelerated aging in just under 40% of individuals. We did not find significant differences in these measures (Table 2). Raw PC test results are also presented in Table 2, as well as the attrition rates of each test performed. Average completion rates were over 95% for both women and men; note that almost half of the women (*N* = 23, 43%) were unable to complete the three-minutes step test (3MST), leading to its early termination (later imputed to the lowest category, see Supplementary Table S2). This could originate from the 30-cm step height, posing a greater challenge to women. The push-up test was also more difficult for women: 28% of women completed two repetitions or less. Note that regardless of any significant differences in performance, reported exertion levels of women and men were comparable.

**Table 2:** Biological age (BA) and physical capacity (PC) measures

		Women		Men		Sex differences^1^
BA (mean ± SD; range)		43.1 ± 5.8; 31.3,54.9		43.8 ± 6.2; 32.8,57.3		*p* = 0.72
∆Age (mean ± SD; range) (% accelerate aging)		− 1.4 ± 5.5; − 12.3,10.3 (36%)		− 0.7 ± 6.4; − 12.2,14.8 (39%)		*p* = 0.77
Physical Capacity		Mean ± SD; range	*N* (completed test %)	Mean ± SD; range	*N* (completed test %)	
Strength	Squats (reps)	36.2 ± 10.4; 15,60.5	53 (100%)	41.3 ± 10.7; 20,70	59 (100%)	***p*** = 0.01
	Push-ups (reps)	7.4 ± 6.1; 0,20	50 (94%)	20.4 ± 10.4; 3,44	59 (100%)	***p* < 0.01**
	Grip (kg)	21.7 ± 6.5; 10.2,36.8	53 (100%)	35.8 ± 8.6; 15.67,50.15	59 (100%)	***p* < 0.01**
	Vertical Jump (cm)	14.3 ± 4.6; 4.4,26.0	49 (93%)	26.5 ± 6.3; 14.7,40.0	56 (95%)	***p* < 0.01**
	Plank (s)	64.7 ± 45.3; 2.8,200	53 (100%)	100.9 ± 43.7; 17,200	57 (97%)	***p* < 0.01**
Flexibility	HBB (cm)	− 4.0 ± 6.3; − 23.5,10.8	53 (100%)	− 8.9 ± 9.1; − 29.5,9.5	59 (100%)	***p* < 0.01**
	Sit and reach (cm)	0.8 ± 9.9; − 20,23	53 (100%)	− 8.8 ± 11.9; − 33,11	59 (100%)	***p* < 0.01**
Endurance	3MST (heart rate)^2^	124.3 ± 15.8; 94.0,153.6	30^4^ (57%)	121.3 ± 18.4; 79.0,170.8	54 (92%)	*p* = 0.43
	Burpees	4.9 ± 4.7; 0,18	51 (96%)	10.4 ± 4.8; 1,21	59 (100%)	***p* < 0.01**
Agility	PTT (s)	16.5 ± 3.7; 11.2,27.1	53 (100%)	14.0 ± 3.5; 9.5,23	59 (100%)	***p* < 0.01**
	FSST (s)	5.8 ± 1.0; 3.6,7.84	53 (100%)	5.7 ± 1.3; 3.4,9.1	59 (100%)	*p* = 0.64
Balance	UPST (s)	27.7 ± 4.6; 12.1–30	53 (100%)	28.1 ± 4.6; 8.4,30	59 (100%)	*p* = 0.51
	UPST blinded (s)	11.5 ± 6.2; 3.1,27.5	53 (100%)	16.2 ± 9.3; 3.2,30	59 (100%)	***p* = 0.02**
Perceived exertion (VAS)		5.0 ± 2.4; 0,9	53 (100%)	5.5 ± 2.4; 0,8	59 (100%)	*p* = 0.23

∆Age the difference between BA and CA (∆Age = BA-CA), *HBB* hand behind back, *3MST* 3 min step test, *PTT* plate tapping test, *FSST* four step square test, *UPST* unipedal stance test, *VAS* visual analogue scale

^1^Differences examined using *t*-test/Mann–Whitney test.

^2^Out of the 23 women that did not complete this test, 21 terminated the test before it ended.

### Correlation between BA and PC measures

In the MAPS cohort, strength, flexibility, endurance, and balance domains were significantly negatively correlated with BA, controlling for CA (Table 3). Since standardization of PC scores accounted for any sex-specific differences, correlations were assessed across the full sample rather than stratifying by sex. We examined the independent contribution of significantly correlated domains (i.e., strength, flexibility, endurance, and balance) to BA using a linear regression model, demonstrating that better performance in endurance, agility, and balance domains was significantly associated with younger BA (Table 4). The logistic regression model used to assess the relationship between ΣPC and a dichotomized ∆Age explained 22% of the variance in participants’ accelerated aging status (Nagelkerke *R*^2^ = 0.22) with an area under the curve of 0.73 (95% CI 0.64–0.83; *p* < 0.001); ΣPC was the only significant variable in the model, with an odds ratio of 0.40 (95% CI 0.25–0.64), showing that each incremental increase in ΣPC corresponded with a 60% reduction of the odds being in an accelerated aging state.

**Table 3:** Pearson’s partial correlation between physical capacity measures (individual tests domain averages, and ΣPC, respectively) and BA, controlling for CA

		Person’s correlation *r*	Significance
Strength domain	Squats	− 0.16	*p* = 0.10
	Push-ups	− 0.30	***p* < 0.01**
	Grip	0.02	*p* = 0.86
	Vertical jump	− 0.15	*p* = 0.12
	Plank	− 0.47	***p* < 0.01**
	Strength domain	− 0.33	***p* < 0.01**
Flexibility domain	HBB	− 0.29	***p* < 0.01**
	Sit and reach	− 0.37	***p* < 0.01**
	Flexibility domain	− 0.44	***p* < 0.01**
Endurance domain	3MST	− 0.45	***p* < 0.01**
	Burpees	− 0.33	***p* < 0.01**
	Endurance domain	− 0.49	***p* < 0.01**
Agility domain	PTT	0.04	*p* = 0.69
	FSST	− 0.15	*p* = 0.12
	Agility domain	− 0.07	*p* = 0.50
Balance domain	UPST	− 0.34	***p* < 0.01**
	UPST blinded	− 0.31	***p* < 0.01**
	Balance domain	− 0.38	***p* < 0.01**
		− 0.49	***p* < 0.01**

Abbreviations: *PC* physical capacity, *BA* biological age, *CA* chronological age, *HBB* hand behind back, *3MST* 3 min step test, *PTT* plate tapping test, *FSST* four step square test, *UPST* unipedal stance test, *ΣPC* composite physical capacity

**Table 4:** A linear regression model examining PC domain association with BA while controlling for CA

	Variable	Coefficients B	Significance (95% CI)
Adjusted *R*^2^ = 0.33, *p* < 0.01	Strength domain	0.08	*p* = 0.47 (− 3.28, 7.12)
	Flexibility domain	− 0.29	*p* < 0.01 (− 9.78, − 2.46)
	Endurance domain	− 0.38	*p* < 0.01 (− 11.17, − 3.45)
	Balance domain	− 0.19	*p* = 0.03 (− 7.71, − 2.46)
	CA	0.03	*p* = 0.73 (− 1.15, 1.63)

*PC* physical capacity, *BA* biological age, *CA* chronological age.

*Agility domain was omitted from this analysis, as it was not significantly correlated with BA.

## Discussion

The aims of this study were to examine the association between an extended, inclusive measure of PC and BA in midlife, as a feasible alternative to the widely reported laboratory biomarkers. This work adds to previous literature by showing that different behavioral marker of physical capacity can be used for assessing health-related body state at midlife [23], transcending previous work estimating BA using limited PC measures in conjunction with laboratory biomarkers [41, 42]. This study addressed the limitations of previously published work by using a comprehensive testing battery, investigating midlife individuals, and quantifying BA using a composite measure. These findings show the link between PC measures, as a possible community-based alternative to laboratory-based methodologies for BA estimation, thus supporting screening of midlife individuals at greater risk for accelerated aging status [43], enabling personalized lifestyle interventions focusing on healthy and active aging [44].

We demonstrate that a PC testing battery could be used for estimating biological aging in midlife healthy individuals: (1) As a composite score, increased PC was associated with younger BA and reduced risk of being in an accelerated aging state; (2) better performance in strength, endurance, balance, and flexibility domains was significantly associated with younger BA, while agility performance was not; (3) subjectively reported exertion levels of women and men were comparable, and total attrition rates were low, suggesting the testing battery, as a whole, was suitable for both sexes and wide ability level.

### Considerations for PC battery

The battery presented here covered a wide range of physical functions, aiming to provide a comprehensive measurement of the physical state of the human body. Observed sex differences in most tests (see Table 2) [45, 46] can be explained by sex-specific muscle mass [30], cardiovascular regulation [47], or even respiratory capacity [48]. These differences support using multiple tests in each domain, to account for personal variation in performance. Some modifications in the battery should be made, adapting the tests for female performance level (i.e., modifying the 3MST and push-up test). Note that the agility tests we used, and the agility domain in general, were not correlated with BA. One possible explanation for this finding is the complexity of agility performance—an interaction of both PC and cognition [49], requiring a different approach when studied in the context of midlife individuals, perhaps calling for the use of tests that are considerably more difficult. Nonetheless, as agility offers insight into certain dimensions of PC, we recommend keeping this domain in future investigations, including more vigorous agility tests, ensuring every aspect of PC is accounted for.

### Midlife as a window of opportunity for healthy-aging promotion interventions

Unveiling the role function plays during midlife is a key to increasing health and lifespan [50, 51]. However, the need for population-wide early detection methods, similar to functional testing batteries designed for older adults (e.g., BESTest[52]), remains largely unanswered. Promotion of healthier aging in midlife is an easier task than curing diseases that have already developed [53], and the suggested testing battery, performed in midlife, can be used both for successfully communicating an easily conveyed functional goal [54] and for monitoring intervention outcomes that may induce a positive change of cardiometabolic state [55]. As such, it could support our efforts to ameliorate the global burden of aging. Additional studies focusing on midlife are required to further establish the contribution of using these markers to inform the public of recommended behavior changes.

### PC as a substitute for BA estimation methods

Many methodologies are currently available for BA estimations [53, 56, 57]. We used a single BA estimation based on a set of blood and physiological biomarkers that were readily available for calculation. KDM-estimated BA is a well-established, effective predictor when it comes to health-function and mortality [41], even when compared with other established methods [53]. The precision of biological clocks depends on sex, as there are differences in life span and health metrics of aging women and men [58, 59]. By showing PC is associated with KDM BA, we place PC as a robust measure for body state, bridging the male–female health-survival paradox [59, 60]. These results suggest that function and performance as health metrics may go beyond the role any specific marker plays in aging mechanism, and reflect on available physical intrinsic capacity [61]. Moreover, identifying the contribution of specific PC domains in this health-related assessment echoes health organizations’ recommendations for adopting active lifestyle, framing PC assessment as a preventive health screening test. Considering that better performance equals younger BA, it enables personalized intervention strategies and allows for easy-to-follow changes in health status that can be monitored even by the patient themselves.

### Strengths and limitations

A major strength of this study is the comprehensive PC testing battery, covering all PC domains that were previously separately associated with aging, while offering a robust measurement across ability levels of normal, healthy, midlife individuals. Although limiting the tests to home-based assessment contributed to the usability of the testing battery, it might ignore biases associated with testing environment, potentially impacting physical performance. Furthermore, we did not account for any selection bias, cognitive, psychological, or sensory capacities that may contribute to BA differences and should be considered in future investigation. Using a single method to estimate BA provides a somewhat narrow perspective on BA, as different methods focus on different aging mechanisms. Larger, population-based studies are needed to further establish the use of PC at a means to estimate BA. Lastly, as this study is limited to cross-sectional data, it does not provide additional information on the role PC plays in predicting BA and accelerated-aging state or offer insights on causal links between the two.

## Conclusions

The suggested PC battery, composed of five PC domains, may offer additional insight on aging state, supporting the identification of midlife individuals at risk for accelerated aging state, while being sensitive enough to reflect different performance ability levels. Specific PC domains and a composite PC score were correlated with BA, emphasizing that behavioral markers could be used to estimate aging state in midlife. Although many studies focus on aging mechanisms to find a cure for “aging disease,” this study emphasizes the importance of identifying individuals at risk for accelerated aging trajectories, supported by the consensus that a lifestyle change can be used to improve health. Clinicians of all professions should assess aging risk and offer personalized behavioral interventions addressing age-related diseases. Physical capacity assessment outcomes can be communicated to the individual tested, not only as an estimate of potential risks, but also as a means of providing a feasible, attainable goal that could be longitudinally monitored.

## Supplementary Information

Below is the link to the electronic supplementary material.

ESM 1 (DOCX 31.9 KB)

## Data Availability

The dataset supporting the conclusions of this article is available in Zenodo repository [10.5281/zenodo.15872110a].

## Abbreviations

BA: Biological age
MAPS: Midlife Aging and Performance Study
CA: Chronological age
SD: Standard deviation
BMI: Body mass index
NHANES: National Health and Nutrition Examination Survey
KDM: Klemera-Doubal Method
ΣPC: Composite physical capacity
ΔAge: The difference between BA and CA
VAS: Visual analogue scale
3MST: 3 Minutes step test
HBB: Hand behind back
PTT: Plate tapping test
FSST: Four-square step test
UPST: Unipedal stance test
