# Supplementary material for: Midlife aging and performance study (MAPS): evaluating biological aging through a physical capacity battery
Source: GeroScience. 2025 Aug 5;48(2):2851–61. doi: 10.1007/s11357-025-01803-6 (PMC12972281; doi:10.1007/s11357-025-01803-6)
Supplement: Supplementary file 1 — (DOCX 31.9 KB) [file 11357_2025_1803_MOESM1_ESM.docx]

**Supplementary Table S1: Detailed description of Methods**

| **Biological age estimation** |
| --- |
| The Klemera-Doubal Method (KDM) algorithm was used to calculate BA based on the following markers: (1) glucose, (2) insulin, (3) high-density lipoproteins, (4) low-density lipoproteins, (5) triglycerides, (6) total cholesterol, (7) body mass index (BMI), (8) diastolic resting blood pressure, (9) systolic resting blood pressure, (10) resting heart rate, and (11) waist circumference. Although this set of biomarkers mainly focuses on cardio-metabolic status, cardio-metabolic aging is known to offer a fair estimation for BA.[1] The BioAge R package utilizes the third United States Health and Nutrition Examination Surveys (NHANES) dataset for training of the KDM algorithm to estimate BA. To simplify, participants’ BA was estimated by the corresponding Chronological Age (CA) at which an individual’s biomarkers would be considered normal. The training set was specified as non-pregnant adults aged 30-50 participating in NHANES-III. This age limit was imposed in the NHANES-III training sample both for comparability with the Midlife Aging and Performance Study (MAPS) age range and to minimize bias from selective survival, which is more prominent at older ages;[2] thus this modification of the published algorithm accounts for age-related changes in the association between specific biomarkers and CA.[3] |
| **Physical capacity estimation** |
| (1) Tests for muscular strength – grip strength,[4] squat test,[5] push-up test,[6] counter-movement vertical jump test,[7] and plank test.[8] These tests have appeared in previous publications and provide an inclusive evaluation for this commonly studied domain.  (2) Tests for cardiorespiratory endurance – three minutes step test (3MST)[9] and burpees test.[10] (3) The flexibility domain was tested using the back stretch (or hand-behind back – HBB) test for upper extremities and the sit and reach test for lower extremities.[11]  (4) Agility tests included the plate tapping test (PTT)[12] and four step square test (FSST)[13] for upper and lower extremities respectively.  (5) The balance domain was assessed using the unipedal stance test (UPST)[14] with eyes open and with eyes closed (“blinded”).  Each test provided a quantitative measure of performance: by force or height units (grip and vertical jump test), heart rate (3MST), time (plank, FSST, PTT, UPST), distance in centimeters (HBB, sit and reach) or a simple count of successful repetitions in one minute (squats, push-ups, burpee).  Throughout the testing session, each test was demonstrated by the examiner before the actual testing; specific tests were skipped if the examiner was concerned for the participant’s safety (e.g., unable to complete previous tests or complaints of nausea or shortness of breath), or if the participant preferred not to participate. The order of performed tests was randomized using five different testing protocol forms, including two fixed resting breaks provided after completion of the more difficult tests (3MST, Burpees, and plank test). Resting breaks were up to 3 minutes long, allowing participants to catch their breath before the testing continued. The total duration to complete all tests was approximately 1 hour. |
| **Strength domain:**   1. **Grip strength** was measured using a Jamar Analogue Hand Dynamometer with participants seated, with their elbows by their sides and flexed to right angles, and with a neutral wrist position. The measure was conducted three times with each hand. The final measure recorded was the average score of the six individual measurements, in kilograms. A higher value represents increased capacity. 2. **The one-minute squat test** was used to measure lower extremity strength. Participants were asked to complete as many squats as possible in one minute. While standing in front of a chair, facing away from it, participants were asked to squat down lightly to touch the chair with the backside before standing back up. This procedure was repeated for one minute or until a participant was unable to continue. Incomplete repetitions were counted as half a repetition; the final measure recorded was the total number of recorded repetitions. A higher number of repetitions represents higher physical capacity. 3. **The one-minute Push up test** was used to measure upper extremity strength. Participants were asked to perform as many push-ups as possible in one minute. Participants started by lying prone with hands shoulder width apart and elbows pointed back. Each repetition required fully extending the elbows while maintaining a straight body posture. Incomplete repetitions were counted as half a repetition. Participants who were unable to perform a single repetition of the test were allowed to perform a modified execution, with their knees on the ground. Modified execution repetition or incomplete repetitions were counted as half-repetitions. A higher number of repetitions represents higher physical capacity. 4. **The counter-movement vertical jump** **test** was conducted to estimate lower extremity explosive strength using a validated smartphone application. In short, each participant was recorded by a smartphone slow-motion video capture, followed by software analysis of recorded vertical jump height performed by analyzing the time spent mid-air while accounting for the participant’s height and weight. Participants were asked to stand with feet positioned shoulder width apart before flexing the knees in a rapid downward motion and extending into the jump. This test was repeated twice, and each recording was analyzed twice using the application. The final measure recorded was the average jump height in centimeters of the four performed recording analyses. A higher jumping height represents higher physical capacity. 5. **The forearm plank test** was used to estimate core muscle strength. Participants assumed a rigid body position so that only their forearms and toes supported their body, and they were instructed to statically hold the position for as long as possible. Brief verbal cues were provided to promote form adherence for test validity. The test continued to a maximal time of 200 seconds or until a participant voluntarily stopped or failed to maintain the proper position (three corrections were allowed prior to terminating the test). The time that the participant succeeded to hold the test posture in seconds, with a maximal score of 200, was recorded. A longer plank hold represents higher physical capacity. |
| **Endurance domain:**   1. **A three-minutes step test (3MST)** was used to estimate maximal oxygen consumption to rate cardiorespiratory fitness. The participant was required to step onto and off a step (30cm height) at an externally set pace (with a metronome) of 96 steps per minutes (e.g., each limb movement counts as a step, counting full stepping on and off the step as four steps) repeatedly for three full minutes, until no longer being able to keep the pace of the metronome, or until reaching a heart rate of 175 bpm. Once stepping was completed, the participant was instructed to sit, and his or her heart rate, measured by a Polar (Polar Electro Oy, Kempele, Finland) H10 heartrate sensor. 2. The sensor data was used to record the participant heart rate at five second intervals for a whole minute. The average heart rate of 12 consecutive records, corresponding to the heart rate of the first minute following the physical effort, was recorded. A lower recorded heart rate represents higher physical capacity. 3. **The one-minute burpee test** is a high intensity motor performance test. Participants were required to perform a series of “burpees” – physical exercises which begins with a squat thrust, followed by a complete push-up, and ending in the standing position. After demonstrating the correct execution of the test, participants were asked to perform as many repetitions as possible in one minute, where incomplete repetitions were counted as half a repetition, and the number of repetitions was recorded. A higher number of repetitions represents higher physical capacity. |
| **Flexibility domain:**   1. **The back scratch test**, or the hand behind back (HBB) test, was used to measure upper extremity flexibility. Each participant stood with one hand behind the back, stretching as far as possible up the spinal column, while stretching and extending the other arm behind the head with the elbow bent, trying to reach the other hand. This was done twice (first with the right hand behind the back and then with the left hand behind the back). The measured distance in centimeters between the middle fingers was recorded; in case the fingers overlapped, the distance was recorded as a positive value; in case the fingers failed to reach, the distance was recorded as a negative value – thus, higher, positive, score was considered better. 2. **The sit-and-reach test** was used to measure lower extremity flexibility. Each participant was seated barefoot on a mat with his or her legs straight, positioned on a wooden box, and asked to reach forward as far as he or she could, beyond the toes and the edge of the box. The measured distance in centimeters between the fingers and the line of the box edge was recorded. Reaching further over the box represented increased physical capacity (positive value), while negative values reflected not being able to reach the edge of the box. |
| **Agility domain:**   1. **The plate tapping test (PTT)** is a part of the EUROFIT test battery, used to measure participants’ upper extremity agility. The participants stood in front of a table, on which the examiner placed a rectangle, 30cm wide, with two yellow discs, 20cm in diameter, positioned to its sides. The non-preferred hand was placed static on the rectangle while the participant was asked to move his or her preferred hand back and forth between the discs over the hand in the middle as quickly as possible. A recording of the time to repeat 25 full cycles (50 taps) was repeated twice. The final measure was the average of the two attempts in seconds. Shorter execution time represents higher physical capacity. 2. **The four-square step test (FSST)** was used to measure lower extremity agility. The test involves four canes positioned to form a “plus” sign on the ground. Participants stood in the first square and were asked to perform four steps by moving over the positioned canes, forward, right, backward, and left, and then return to the starting position in reverse, thus performing the procedure with a total of four steps clockwise followed by four steps counterclockwise, as fast as possible. The final measure was the average of two attempts, measured in seconds. A shorter execution time represents higher physical capacity. |
| **Balance domain:**   1. **The unipedal stance test (UPST)** was used to measure static balance. Participants were asked to stand on one leg, while the opposite heel was placed over the standing knee joint. This position was maintained for 30 seconds. If the participant failed, a second attempt was initiated. The final measure was the average best time in seconds of maintained balance of each leg (i.e., average of the best attempt for the right leg and the best attempt for the left leg). A longer duration of balance represents higher physical capacity. 2. **The unipedal stance test (UPST)** was performed again, with eyes closed. The above procedure was repeated for both sides with eyes closed, and the scoring was the same. |

**Supplementary Table S2: Composite physical capacity calculations**

| 1 | Composite scores were calculated by rescaling each measure based on rank and percentiles, to a 0 (low ability, value<25^th^ percentile) 0.33 (low-average ability, value range 25^th^-50^th^ percentile), 0.66 (high-average ability, value range 50^th^-75^th^ percentile) or 1 (high ability, value>75^th^ percentile). |
| --- | --- |
| 2 | For UPST, a variable that was negatively skewed (25^th^, 50^th^, and 75^th^ percentiles were both the same), different cut scores were included, based on clinical expertise. 30 seconds were scored as 1, 26 to 29 seconds were scored as 0.66, 21 to 25 seconds were scored 0.33 and a result under 21 seconds was scored a 0. |
| 3 | For 3MST, due to high attrition rates specifically in women participants, inability to complete the test was imputed to a 0, reflecting the lower capacity of participants unable to complete it (23 women, 5 men). |
| 4 | For vertical jump test, missing scores due to poor video capture or technical issues, were imputed to a 0.5 (4 women, 3 men). |
| 5 | Domain-specific scores were calculated by averaging the scores of all tests of a specific domain for a domain composite score of 0 to 1; the final ΣPC was calculated by aggregating the scores of all 5 domains, to a total score ranging from 0 to 5, thus each domain was equally weighed in the final ΣPC score. |

**Supplementary references**

1. Lind L, Ingelsson E, Sundström J, Siegbahn A, Lampa E. Methylation‐based estimated biological age and cardiovascular disease. European Journal of Clinical Investigation. 2018;48(2):e12872. doi: 10.1111/eci.12872.

2. Kwon D, Belsky DW. A toolkit for quantification of biological age from blood chemistry and organ function test data: BioAge. Geroscience. 2021;43(6):2795-808. doi: 10.1007/s11357-021-00480-5.

3. Shapiro I, Belsky DW, Israel S, Youssim I, Friedlander Y, Hochner H. Familial aggregation of the aging process: biological age measured in young adult offspring as a predictor of parental mortality. GeroScience. 2023;45(2):901-13. doi: 10.1007/s11357-022-00687-0.

4. Massy-Westropp NM, Gill TK, Taylor AW, Bohannon RW, Hill CL. Hand Grip Strength: age and gender stratified normative data in a population-based study. BMC research notes. 2011;4(1):1-5. doi: <https://doi.org/10.1186/1756-0500-4-127>.

5. Fry AC, Kudrna RA, Falvo MJ, Bloomer RJ, Moore CA, Schilling BK, et al. Kansas squat test: A reliable indicator of short-term anaerobic power. The Journal of Strength & Conditioning Research. 2014;28(3):630-5. doi: <https://doi.org/10.1519/jsc.0b013e3182a0cb23>.

6. Baumgartner TA, Oh S, Chung H, Hales D. Objectivity, reliability, and validity for a revised push-up test protocol. Measurement in Physical Education and Exercise Science. 2002;6(4):225-42. doi: <https://doi.org/10.1207/S15327841MPEE0604_2>.

7. Bogataj Š, Pajek M, Hadžić V, Andrašić S, Padulo J, Trajković N. Validity, reliability, and usefulness of My Jump 2 App for measuring vertical jump in primary school children. International journal of environmental research and public health. 2020;17(10):3708. doi: <https://doi.org/10.3390%2Fijerph17103708>.

8. Strand SL, Hjelm J, Shoepe TC, Fajardo MA. Norms for an isometric muscle endurance test. Journal of human kinetics. 2014;40:93. doi: <https://doi.org/10.2478%2Fhukin-2014-0011>.

9. Beutner F, Ubrich R, Zachariae S, Engel C, Sandri M, Teren A, et al. Validation of a brief step-test protocol for estimation of peak oxygen uptake. European journal of preventive cardiology. 2015;22(4):503-12. doi: <https://doi.org/10.1177/2047487314533216>.

10. Podstawski R, Markowski P, Clark CC, Choszcz D, Ihász F, Stojiljković S, et al. International Standards for the 3‐Minute Burpee Test: High‐Intensity Motor Performance. Journal of human kinetics. 2019;69:137. doi: <https://doi.org/10.2478%2Fhukin-2019-0021>.

11. del Pozo-Cruz B, Gusi N, Adsuar JC, del Pozo-Cruz J, Parraca JA, Hernandez-Mocholí M. Musculoskeletal fitness and health-related quality of life characteristics among sedentary office workers affected by sub-acute, non-specific low back pain: a cross-sectional study. Physiotherapy. 2013;99(3):194-200. doi: <https://doi.org/10.1016/j.physio.2012.06.006>.

12. Adam C, Klissouras V, Ravazzolo M, Renson R, Tuxworth W, Kemper H, et al. EUROFIT-European test of physical fitness. 1 ed. Rome: Council of Europe, 1987; 1987.

13. Torlak F, Moffat M. PP17 Four square step test normative data for healthy young adults. British Journal of Sports Medicine; 2014.

14. Springer BA, Marin R, Cyhan T, Roberts H, Gill NW. Normative values for the unipedal stance test with eyes open and closed. Journal of geriatric physical therapy. 2007;30(1):8-15. doi: <https://doi.org/10.1519/00139143-200704000-00003>.
